# GALA: group analysis leads to accuracy, a novel approach for solving the inverse problem in exploratory analysis of group MEG recordings

**DOI:** 10.3389/fnins.2015.00107

**Published:** 2015-04-21

**Authors:** Vladimir V. Kozunov, Alexei Ossadtchi

**Affiliations:** ^1^MEG Centre, Moscow State University of Psychology and EducationMoscow, Russia; ^2^Centre for Cognition and Decision Making, National Research University Higher School of EconomicsMoscow, Russia; ^3^Laboratory of Control of Complex Systems, Institute of Problems of Mechanical Engineering Russian Academy of SciencesSt. Petersburg, Russia

**Keywords:** MEG, inverse problem, group analysis, rank of leadfield matrix, covariance model, maximum likelihood, iterations

## Abstract

Although MEG/EEG signals are highly variable between subjects, they allow characterizing systematic changes of cortical activity in both space and time. Traditionally a two-step procedure is used. The first step is a transition from sensor to source space by the means of solving an ill-posed inverse problem for each subject individually. The second is mapping of cortical regions consistently active across subjects. In practice the first step often leads to a set of active cortical regions whose location and timecourses display a great amount of interindividual variability hindering the subsequent group analysis. We propose Group Analysis Leads to Accuracy (GALA)—a solution that combines the two steps into one. GALA takes advantage of individual variations of cortical geometry and sensor locations. It exploits the ensuing variability in electromagnetic forward model as a source of additional information. We assume that for different subjects functionally identical cortical regions are located in close proximity and partially overlap and their timecourses are correlated. This relaxed similarity constraint on the inverse solution can be expressed within a probabilistic framework, allowing for an iterative algorithm solving the inverse problem jointly for all subjects. A systematic simulation study showed that GALA, as compared with the standard min-norm approach, improves accuracy of true activity recovery, when accuracy is assessed both in terms of spatial proximity of the estimated and true activations and correct specification of spatial extent of the activated regions. This improvement obtained without using any noise normalization techniques for both solutions, preserved for a wide range of between-subject variations in both spatial and temporal features of regional activation. The corresponding activation timecourses exhibit significantly higher similarity across subjects. Similar results were obtained for a real MEG dataset of face-specific evoked responses.

## 1. Introduction

Nowadays, magnetoencephalography (MEG) offers a unique opportunity for non-invasive time-resolved exploration of neuronal processes taking place in the human brain. MEG, as well as EEG, directly register electrical processes and thus provide for a significantly higher temporal resolution as opposed to other non-invasive methods registering indirect correlates of neuronal activity, such as, for instance, fMRI and PET. Produced by neuronal sources quasi-static magnetic field outside the head is significantly less sensitive to the anisotropy of conductive properties of the head tissues than the electric potential on the scalp. Therefore, in contrast to EEG, MEG enjoys a simpler and more accurate volume conductor model (Sarvas, 1987) linking geometric properties of a neuronal source to sensor signals.

### 1.1. Preliminaries

In order to localize neuronal sources using the non-invasive measurements one has to solve the ill-posed electromagnetic inverse problem. The main challenge in interpreting the inverse solution is to distinguish between the true cortical activity and that brought in by the associated non-uniqueness of the inverse problem in MEG(EEG). The exact shape of such a spread varies greatly across subjects as a result of its direct dependence on the forward model operator taking into account individual highly variable cortical surface geometry. Thus, due to high variability of anatomic characteristics and geometric properties of the experimental session, practical application of such a strategy results in solutions that vary greatly across individual subjects. This strategy makes it possible to detect cortical regions whose activation exhibits statistically significant differences between experimental conditions within short time-window (e.g., statistical parametric mapping). However, the whole timecourse profiles of activation estimated for these regions could be so dissimilar between subjects that it is impossible to unambiguous explore any of its characteristics in time domain (e.g., connectivity analysis).

One way around this problem is to use parametric methods to solve the inverse problem and find a finite set of dipoles (location, orientation, timecourse) that explain the large proportion of variance in the data see (Tanskanen et al., 2005; Deffke et al., 2007; Schweinberger et al., 2007; Wengenroth et al., 2014; Woodhead et al., 2014) as examples. This approach allows (although for a limited extent) exploratory data analysis in contrast to approaches in which ROIs are predefined (and not data driven). The drawback of this technique lies in low accuracy of multidipolar fits in a more than 2-dipole case and the difficulty of *apriori* determination of the number of dipoles to fit (see however, Mosher and Leahy, 1999) as well the cross-subject co-registration issues. In principle, the approaches similar to those exercised in Darvas et al. (2005) can be used to further advance multi-subject multi-dipole analysis.

Another way around lies in using extended ROIs with borders defined based on the functional properties via statistical analysis (Pantazis et al., 2005; Litvak et al., 2011) of the data within some prespecified time window (Gross et al., 2007; Altamura et al., 2010; Brang et al., 2010; Clarke et al., 2011; Lee et al., 2011). In order to avoid double dipping problem (Kriegeskorte et al., 2009) separate data sets for determining the ROIs and ROI centered analysis need to be used (Brang et al., 2010), which significantly complicates a study. The problem is that even this procedure does not guarantee reliable detection of ROI borders. Activation profiles of spatially extended regions can be hardly modeled by a single time course. Regions showing sufficiently high contrast may be too focal to allow justifiable co-registration across subjects. Also, non data-driven ROI specification completely excludes the possibility of detailed spatial exploratory analysis.

Group analysis is one of the main approaches used in cognitive neuroscience to account for between-subject variability. However, matching ROIs from several subjects constitutes an additional challenge as even if the exact correspondence between meshes is established the data-driven approach described above does not guarantee significant intersection of the corresponding ROIs across subjects and the similarity of activation timeseries is not enforced as well. The described problems do not preclude from efficient studies of within subject connectivity and other statistical properties of activations dynamics. However, significant across-subject variation of activation profiles may become a serious obstacle in interpretation of results on the group level.

Consequently, the goal of this work was to develop a method for solving the inverse problem at a group level that would increase the accuracy of spatial localization of a set of simultaneously active cortical regions and allowed for more accurate estimation of the corresponding activation timeseries. Another important problem that needs to be solved is that of establishing the correspondence of distinct ROIs across subjects.

The solution that we provide attempts to solve the two problems simultaneously within a probabilistic framework and thus allows us to significantly improve on the existing approaches that perform the two tasks separately (first localize then match). In contrast to many other methods the proposed technique takes advantage of individual variations of cortical geometry and head position inside the helmet. Our method exploits across subject variability as the source of additional information to deliver more accurate decomposition of the non-invasively observed activity into a set of functionally relevant components. These conceptual innovations allow us to achieve more accurate solutions for each of the two problems. To solve an under-determined inverse problem, modeling assumptions about the solution must be made. The accuracy of a solution is crucially dependent on the plausibility of these assumptions. Earlier attempts to benefit from between-subject variation of forward models were made at the post individual inverse stage (Larson et al., 2014) which only partly exploits the potential for improvement of localization accuracy.

### 1.2. Modeling assumptions and approach outline

In electrophysiology the vast number of data analysis are based on the assumption of similarity in measured responses across subjects. The most revealing example is the concept of grand average across subjects. Data from different subjects for one experimental paradigm get averaged in the attempt to obtain the response corresponding exactly to the process in study and discard any individual variability. Here we suggest to use these similarities as modeling assumptions or prior knowledge to constrain solution of the inverse problem within the newly introduced group-level algebraic inverse paradigm.

In the approach presented here there are two main assumptions. The first one is that functionally equivalent brain activities for different subjects are located in the same anatomical structures that may not exactly coincide on the cortex for different subjects but are, nevertheless, quite close to each other. The second assumption is that these activities for different subjects unfold in time in very similar ways so that the corresponding time courses are correlated. These assumptions of ours are based on the concept of modularity of brain organization that is a cornerstone of most modern brain theories (Fodor, 1983). The assumption about similarity (not exact) of anatomical structure and location of functionally homologous brain regions appears to be quite plausible, at least for the experiments with normal adult subjects (Cosmides and Tooby, 1994). Feasibility of the second assumption stems from a large number of studies that with the use of subdural grid and depth electrode recordings (e.g., Halgren et al., 1980; Smith et al., 1986; Friederici et al., 1999) show that there is rather reproducible sequence of characteristic features of time courses such as peak latencies, extent of positive or negative excursions for most subjects in response to a fixed stimulus. These findings allow us to expect reasonably high correlation of activation timeseries across individuals (Clune et al., 2013). As we show in this work, the explicit constraint on the across-subject similarity of activations improves the accuracy of the inverse solution.

## 2. Materials and methods

### 2.1. Algorithm

To facilitate constraints on across-subject level and allow for anatomically informed source reconstruction, first of all, the exact match between cortices of different subjects must be established. The canonical mesh (Mattout et al., 2007) ensures that activity is reconstructed in the same source space over subjects (Talairach and Tournoux, 1988). Briefly, the forward model for each subject starts with a template mesh, defining a lattice of sources on the cortical surface. This mesh is then warped using an inverse spatial normalization so that the resulting canonical mesh is in the same place as the subject's cortical sheet. After warping, subject specific forward fields (i.e., a gain matrix) are computed using standard electromagnetic forward modeling procedure using a single-shell head model. Reconstruction of the canonical sources corresponds to the inversion of these forward models, given some data. After inversion of the ensuing forward model, reconstructed activity can be assigned to the same mesh vertices over subjects.

Consider a set of observation equations for each subject

(1) $$Y_{i}=L_{i}J_{i}+\varepsilon_{i}$$

where for the *i*-th out of total *N* subjects *Y_i_* ∈ 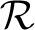
^c×t^—measurement vector, *L_i_* ∈ 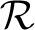
^c×n^—*i*-th subject lead field matrix, calculated on the basis of individual anatomy image, and *J_i_* ∈ 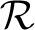
^n×t^ is a vector of amplitudes of *n* current dipoles distributed inside the gray matter of the *i*−th subject's brain with fixed orientations normal to the cortical surface, *n* is the same for all *N* subjects and the necessary correspondence between vertices across subjects is ensured by a coregistration mechanism (e.g., canonical mesh), ε*_i_* ∈ 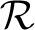
^c×t^—measurement noise vector, *c*—number of channels, *t*—number of timeslices. Having exact correspondence between sources (mesh vertices) of different subjects we can try to express the assumptions described above in a very simple form

(2) $$\left[\begin{array}{c} Y_{1}\\ \vdots \\ Y_{N} \end{array}\right]=\left[\begin{array}{c} L_{1}\\ \vdots \\ L_{N} \end{array}\right]\bar{J}+\left[\begin{array}{c} \varepsilon_{1}\\ \vdots \\ \varepsilon_{N} \end{array}\right]$$

where *J*- common vector of activations for all subjects *J* ∈ 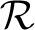
^n×t^.

Unfortunately, the formulation is not realistic as it is impossible to really justify the fundamental assumption of exact deterministic across-subject equivalence of activation vectors stipulated by formulation in Equation (2). Even with established match between all individual vertices over subjects this correspondence is exact only in the anatomical but not in the functional sense. Real functional ROIs can be close to each other for different subjects but overlap only partly or not overlap at all. Similar arguments can be made about correlation of electrical activity of the corresponding sources. To implement this we need to relax the constraints imposed by the use of common vector of activations in Equation (2) which naturally leads to the second form of the group data model that allows to impose similarity constraints using probabilistic language

(3) $$\left[\begin{array}{c} Y_{1}\\ \vdots \\ Y_{N} \end{array}\right]=\left[\begin{array}{ccc} L_{1} & \cdots & 0\\ \vdots & \ddots & \vdots \\ 0 & \cdots & L_{N} \end{array}\right]\left[\begin{array}{c} J_{1}\\ \vdots \\ J_{N} \end{array}\right]+\left[\begin{array}{c} \varepsilon_{1}\\ \vdots \\ \varepsilon_{N} \end{array}\right]$$

A concise form of this equation

(4) Y=LJ+ε

can be obtained with the following notations *Y* = [*Y*^*T*^_1_, …, *Y^T^_N_*]*^T^, Y* ∈ 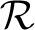
^Nc×t^ and a group-wise source activations vector *J* = [*J^T^*_1_, …, *J^T^_N_*]^*T*^, with *J* ∈ 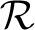
^Nn×t^. Block-diagonal matrix

$L=\left[\begin{array}{ccc} L_{1} & \cdots & 0\\ \vdots & \ddots & \vdots \\ 0 & \cdots & L_{N} \end{array}\right],$ *L* ∈ 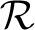
^Nc×Nn^ having blocks of subject's lead field matrices *L_i_* ∈ 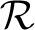
^c×n^ provides the same linear relationship between data and sources as in the single subject case.

Theoretically, a solution to this problem can be found within maximum *a posteriori* probability framework that in addition to likelihood term requires a prior term expressing the desired properties of the solution to be found. For example, to formalize the requirement of exact coincidence of individual activations the following source space prior *p*(*J|C*) ∝ exp(−$\frac{1}{2}$*J^T^C*^−1^*J*) can be used with *C* ∈ 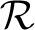
^Nn×Nn^ of the form

(5) $$\left[\begin{array}{ccc} I & \cdots & I\\ \vdots & \ddots & \vdots \\ I & \cdots & I \end{array}\right]$$

where *I* ∈ 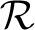
^n×n^ is an *n* × *n* identity matrix. The technical fact that *C* is not invertible requires considering this prior in the generalized delta-function sense via the limit *C*^−1^ = lim_ϵ→0_ (*C* + ϵ*I*)^−1^. With ϵ → 0 Equations (2) and (3) become equivalent, as the specified covariance expresses the requirement that *cov*(*J_i_, J_j_*) = 1 corresponding to the exact equivalence of individual activations.

**Figure 1:**
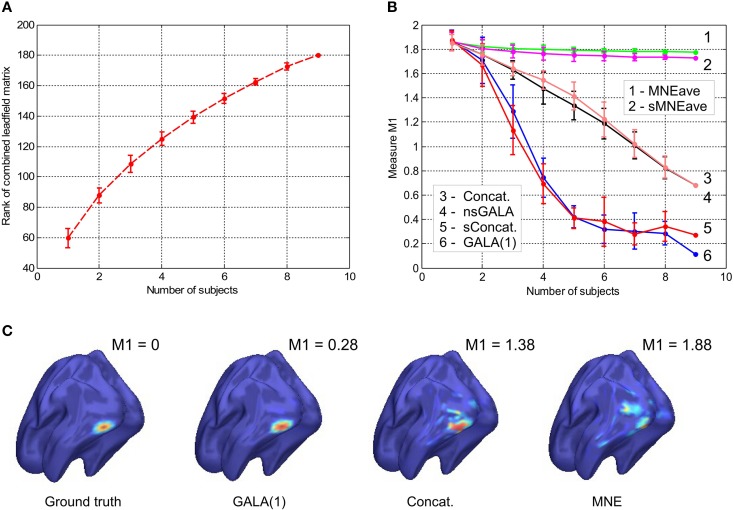
**(A)** Rank of combined leadfield matrix as a function of the number of lead field matrices concatenated. We show results for condition number threshold of *e*^−8^. We observe significant growth of the effective rank. The observed growth of the group model rank illustrates the amount of additional information brought into the inverse problem by considering all subjects simultaneously and ensures potentially increased accuracy in the associated inverse problem solutions. **(B)** Dependence of *M*1 accuracy of ground truth activity recovery measure on the number of subjects in the group inverse for six different methods (lower value of *M*1 corresponds to higher accuracy). To simulate the data we used the ground-truth patch as shown in **(C)**. **(C)** To appreciate qualitative picture corresponding to different values of *M*1 consider the following localization results of the simulated cortical activity. From left to right: ground truth, GALA(1) on 6 simulated subjects, lead-field concatenation without smoothness constraint (Concat.); MNE w/o averaging (MNE).

These (largely equivalent) forms Equations (2, 3) help to highlight different features of the approach suggested here. The first form Equation (2) most clearly represents the main advantage of GALA, namely adopting additional anatomical information used to solve the inverse problem. Indeed, the stacked gain matrix as it is used in Equation (2) with a common vector of activations for all subjects *J*, results into significantly less under-determined problem than any of the individual ones because due to geometric and anatomical reasons *rank*(*L*) ≥ *rank*(*L_i_*) for any *i*, for a quantitative support of this statement see Figure 1A in Section 3. Note, that this characteristic of GALA differs it from the earlier suggested approaches for group inversion (Litvak and Friston, 2008; Henson et al., 2011) based on finding common source space for all subjects. The use of such a common subspace approach leads to the ultimate decrease of the group forward model rank as compared to the individual models. The more disparate individual forward models are the more pronounced rank decrease is. For the GALA approach suggested here the situation is exactly the opposite, as the addition of any new subject increases the rank of *L* and improves solution's accuracy.

The second form of the group data model expressed by Equation (3) allows the use of composite source space covariance priors to impose adequate constraints onto the group inverse. We will show that adding the simplest diagonal covariance component allows to relax the unrealistic constraints imposed by the use of common vector of activations in Equation (2).

Here, we stick to Bayesian approach and assume that *J* and ε in Equation (4) follow multivariate Gaussian distributions *p*(*J*) ∝ 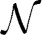
(*J; 0, Q*) and *p*(ε) ∝ 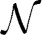
(ε; *0*, Σ_ε_) where *Q* and Σ_ε_ are covariance matrices of source activations and noise respectively.

According to the well established framework (Wipf and Nagarajan, 2009) the posterior distribution of the source activity *J* given data *Y* can be derived from Bayes theorem as

(6) $$p(J|Y)=\frac{p(Y|J)p(J)}{p(Y)}$$

(7) $$\begin{array}{l} p(J|Y)\propto p(Y|J)p(J)\propto \exp(-{\left(LJ-Y\right)}^{T}\\ \;\Sigma_{\varepsilon }^{-1}(LJ-Y)-J^{T}Q^{-1}J) \end{array}$$

So that if *Q* is known, estimated activity *Ĵ* can be obtained as

(8) $$\hat{J}=QL^{T}{\left(\Sigma_{\varepsilon }+LQL^{T}\right)}^{-1}Y$$

However, since *Q* is not known, a suitable approximation $\hat{Q}$ must first be found. As suggested in Phillips et al. (2005) we adopt the following parametrization

(9) $$Q=\sum_{i\;=\;1}^{N_{q}}h_{i}C_{i}$$

where *C_i_*, *C_i_* ∈ 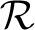
^Nn×Nn^ are known matrices which impose constraints on the structure of source covariance matrices and *h_i_* are non-negative unknown parameters (sometimes referred to as hyperparameters, Friston, 2002) and should be derived from the data. As long as basis set of covariance matrices in Equation (9) is fixed the common way to estimate hyperparameters is based on integrating out the source activities *J* and considering hyperparameters as the only parameters. Posterior probability of hyperparameters from Bayes theorem is *p*(*h|Y*) ∝ *p*(*Y|h*)*p*(*h*). In the case of flat priors on hyperparameters we can write that *p*(*h|Y*) ∝ *p*(*Y|h*).

This gives *p*(*Y|h*) ∝ 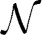
(*Y; 0*, Σ_*y*_) ∝ |Σ*_y_*|^−$\frac{1}{2}$^exp(−$\frac{1}{2}$*Y^T^*Σ*^−1^_y_Y*) where Σ_*y*_ = Σ_ε_ + *LQL^T^* is sensor space sample covariance corresponding to the prior source covariance.

Finding hyperparameters that maximize *p*(*Y|h*) is equivalent to maximizing the corresponding ML cost function 2ln(*p*(*Y|h*)) = −*trace*[*C_y_*Σ^−1^*_y_*] − ln|Σ_*y*_| where *C_y_* = *YY^T^* is the empirical covariance. This procedure is sometimes referred to as type-II maximum likelihood (Wipf et al., 2010).

Our key contribution is in proposing novel iteratively adjusted parametric covariance structure of *Q* that on the one hand imposes the desired similarity between subjects and on the other hand models subject specific activity and thus prevents the distortion of the common (across subjects) part of the solution. Identification of this covariance model is implemented via iterative partitioning of the source space into two complementary sets. The first set Θ is a set of vertices whose activations are similar across the group of *N* subjects. The complementary set Δ is defined correspondingly as Δ = Ω − Θ where Ω is a set of all vertices of all *N* subjects.

**Figure 2:**
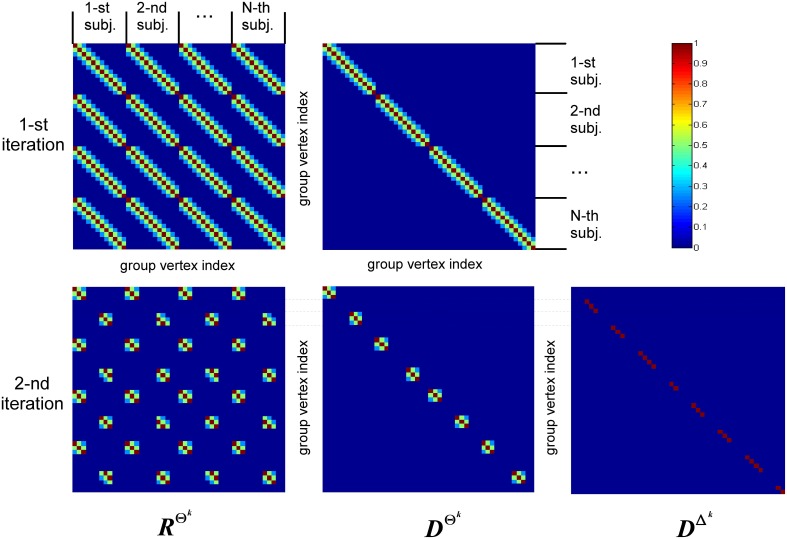
**This diagram illustrates covariance matrix components as described in Algorithm section**. *R*^Θ^*k*^^ is the covariance matrix on the k-th iteration modeling similarity across neighborhood vertices both within one subject and across them. For visualization purposes this matrix has been obtained from the identity matrix convolved with the Gaussian kernel, corresponding to the simplified neighborhood system determined by linear voxel index. We have also significantly reduced each subject's vertex count. *D*^Θ^*k*^^ is the covariance matrix modeling across-subject variability of activity within common activity regions. *D*^Δ^*k*^^ appears starting from the second iteration and models individual activity outside common activity regions. Note, non-zero elements of this matrix correspond to the vertices in which *R*^Θ^*k*^^ and *D*^Θ^*k*^^ components have zero entries, reflecting the fact that set Δ^*k*^ is complementary to set Θ^*k*^.

The structure of covariance matrix as composed of three types of components is shown in Figure 2. Here we illustrate our covariance modeling approach for the simplified neighborhood system determined by linear voxel index. *R*^Θ^*k*^^ is the covariance matrix component on the *k*-th iteration modeling similarity across neighborhood vertices both within one subject and across them. *D*^Θ^*k*^^ is the covariance matrix modeling across-subject variability of activations within common activity regions as well as the variability of activation power of different ROIs within one subject. *D*^Δ^*k*^^ appears starting from the second iteration and models individual activations outside the common activity regions. Note, that since Δ and Θ are complementary subsets of Ω, the non-zero elements of *D*^Δ^*k*^^ correspond to the vertices in which *R*^Θ^*k*^^ and *D*^Θ^*k*^^ have zero entries. Thus, on the *k*−th iteration our group-wise data covariance matrix is parametrized as

(10) $$\Sigma_{y}^{k}=h_{1}^{k}\Sigma_{\varepsilon }+LQ^{k}L^{T}$$

with

(11) $$Q^{k}\;=\;h_{2}^{k}R^{\Theta^{k}}\;+\;h_{3}^{k}D^{\Theta^{k}}\;+\;\sum_{j\;=\;2}^{k}h_{2+j}^{k}D^{\Delta^{j}}$$

Here, superscripts Θ^*k*^ and Δ^*k*^ specify the set of vertices whose covariance has the corresponding component. The term *h^k^*_1_Σ_ε_ corresponds to sensor noise covariance. In general, matrix Σ_ε_ is a *Nc* × *Nc* block diagonal matrix that can be built from the noise covariance matrices for each subject. In this work we used scaled identity matrix to represent sensor noise covariance.

The first term in Equation (11) expresses the requirement that each source of every subject should co-vary with the corresponding sources of other subjects. This spatial correspondence is defined in a smooth way using the Gaussian kernel weighted neighborhood on a cortical mesh. This is implemented using matrix *R*^Θ*^k^*^ = {*r^Θ^k^^_ij_*}, a symmetric matrix modeling similarity across neighborhood vertices both within one subject and across them. The best way to introduce *R*^Θ^*k*^^ is to represent it as a convolution of two parts emphasizing it characteristics. Consider a matrix composed of *N* × *N* identity matrices *I, I* ⊂ 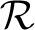
^n×n^ Equation (5), stating that activity in every vertex of any subject exactly co-varies with that of the corresponding vertices of other subjects, and convolve it with discrete Gaussian kernel 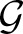
(*d*) = *exp*(−*d*^2^), where *d*—is normalized geodesic distance in adjacency metric on the mesh, so that the closer the neighbor vertices are to the center of the kernel the more their activity is expected to coincide with that of the central voxel. Formally the elements of matrix *R*^Θ^*k*^^ can be represented as

(12) $$r_{i+pn,j+pn}^{\Theta^{k}}=\left\{ \begin{array}{l} \exp(-d_{ij}^{2}),\;\text{for}j\in O_{i},\;i+pn\in \Theta^{k},\;j+pn\in \Theta^{k}\\ 0,\text{ otherwise} \end{array}\right.$$

for *i* = 1, …, *n*; *j* = 1, …, *n*; *p* = 0, ….*N* − 1 where *O_i_* is a set of vertices in the neighborhood of the i-th vertex defined by thresholding the kernel 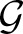 and *d_ij_* is cortical distance in adjacency metric between the i-th and the j-th vertices. Please, refer to Figure 2 for a simplified graphical representation of this matrix.

Despite Gaussian smoothing characteristics of matrix *R*^Θ^*k*^^ its structure forces source activity values to be exactly the same for corresponding vertices of every subject. To relax this constraint the second component is needed. It models independent variations of sources for the same set Θ^*k*^ and is formalized as matrix *D*^Θ^*k*^^, see Equation (11). Matrix *D*^Θ^*k*^^ is an *Nn* × *Nn* diagonal matrix in which we first set to unity only the elements corresponding to the indices in set Θ^*k*^ and then convolve the resultant diagonal matrix with the same Gaussian kernel 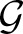
(*d*). Unlike the first covariance basis element, *D*^Θ^*k*^^ does not impose cross-subject similarity and allows for individual variations of the evoked activity. The main purpose of hyperparameters estimation then is balancing between the similarity and independence of activity of the corresponding sources across subjects.

The last term in Equation (11) appears starting from the second iteration and has a composite structure. Sequential iterations reparametrize it by adding at each iteration a new matrix corresponding to the new complementary set Δ^*k*^. This component gathers the activity that is least similar across subjects, and thus its terms are created on the basis of *Nn* × *Nn* identity matrices with non-zero elements corresponding to the vertices in set Δ_*k*_.

So unlike the second component that models across-subject variability of response in the commonly active regions, this third component describes individual activity that does not have any similarity across-subjects and therefore is not of interest. Based on these considerations we term this activity as source noise. However, while the activity of vertices in set Δ^*k*^ should be excluded from the subsequent analysis we have to still account for it in the covariance structure to prevent the distortion of the common part of the solution and avoid leaking of this individual activity into the common part of the solution.

In a nutshell, the GALA algorithm starts assuming the ideal scenario that all the vertices of all subjects belong to a set Θ, i.e., Θ^1^ = Ω and Δ^1^ = 0 and proceeds iteratively. At the k-th iteration, using covariance matrix from the previous, k-1-st iteration we find hyperparameters *h_i_, i* = 1, …, *k* + 2 and the source vector estimate *Ĵ^k^*. Then, based on the estimate of *Ĵ^k^* we adjust the partitioning of vertices between sets Θ*^k^* and Δ*^k^* keeping it so that set Θ^*k*^ contains only half of the best vertices from set Θ^*k*−1^. The worst half goes to Δ^*k*^ set. Having obtained this new partition we reparametrize our source space covariance matrix *Q^k^* according to Equation (11) and perform the new iteration.

For reparametrization we use 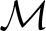
*_i_* = ∑_*j*∈Ψ_
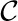
*^J^k^^_ij_, i* = 1, …, *Nn* as the criterion for selection of the best vertices. In this expression *C^J^k^^_ij_* = *cov* (*Ĵ^k^_i_*(*t*), *Ĵ^k^_j_*(*t*)) reflects similarity of the *i*-th and *j*-th vertices activation timeseries on the k-th iteration, *t* in brackets indicates that covariance is calculated over time dimension, and set Ψ contains only those indices for which *R^Θ^k^^_ij_* > 0. This measure defines for each vertex the value which combines its variance with the degree of similarity of its time course and that of the nearest neighbors inside *O_i_* both within one subject and across subjects as indicated by non-zero entries of *R^Θ^k^^*.

**Figure 3:**
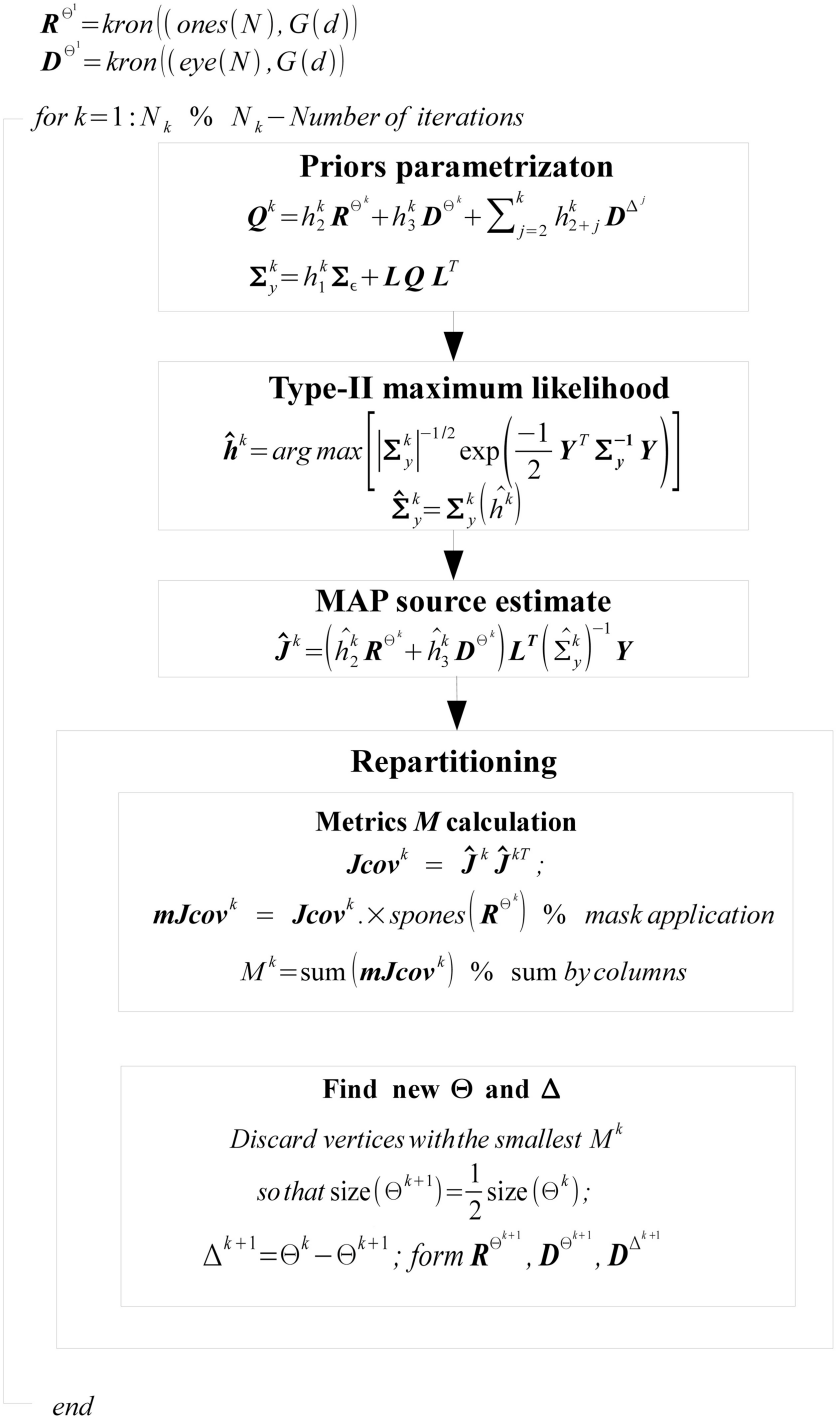
**Diagram of GALA workflow**. Variable names match those in the text.

We summarize the overall work-flow in the diagram on Figure 3.

### 2.2. Methods of comparison

GALA was designed to cope with deviations from the standard assumption of common activations vector across subjects in the group MEG data. Each of the simulation studies described in Sections 3.1.1–3.1.4 were designed to study GALA's performance as a function of particular parameter characterizing such deviation. When describing the simulations, each section adds the specific details that pertain to the particular study.

In the present study we constrained the sources to lie on a tessellated mesh of the cortical mantle. The sources are considered as dipoles with fixed orientations normal to the local curvature of the mesh. The meshes were obtained on the basis of high-resolution structural T1-weighted MRIs acquired on a 1.5T Toshiba ExcelArt Vantage scanner (*TR* = 12 ms, *TE* = 5 ms, flip angle = 20°, slice thickness = 1.0 mm, voxel size = 1.0 × 1.0 × 1.0 mm3).

Purely for simplicity purposes in this work we used 204 planar gradiometer sensors arranged according to the design implemented in Elekta Neuromag Vector View 306 channel MEG system. We used single-shell forward model to calculate magnetic field in a realistic volume conductor (Nolte and Curio, 1997). For each subject we computed orientation constrained forward models for two different meshes we used to produce simulated data (8196 vertices) and to solve the inverse problem (5124 vertices). This was done to mimic realistic conditions.

#### 2.2.1. Simulated data

Here we outline the general procedure for generating simulated datasets used in this study.

For each simulated patch we first choose a vertex to serve as a gravity center for the group of corresponding patches in all subjects. To model variations of patch location across subjects we specified central vertices for individual patches by varying the position of this center of gravity vertex. To form individual patch we then selected vertices within pre-specified distance from the individual central vertex.

Patch activations, modeling evoked response, were created by alterations of the basic activation function. As the basic function we chose sine of square root of time index modulated by the generalized bell-shaped Matlab function. The use of square root in the argument of the sine function provided the increase of oscillation period with increased latency of the response. Bell-shaped function parameters were chosen to provide 2–3 half-waves in the response. Then, to model across-subject variations we added the parameters responsible for response jitter and additional spread. In all the simulations we used evoked fields and therefore, the reported SNR values correspond to that of the trial-averaged evoked fields and not single epoch responses. To simulate sensor space data we then multiplied subject vertex activation timeseries by the corresponding lead field matrices as dictated by the observation Equation (1).

To assess the quality of inverse solutions we used two indicators separately reflecting localization accuracy and the precision of activation timeseries recovery. We used independent of any threshold measure *M*1 calculated as the sum of vertex by vertex absolute differences of the normalized estimate and ground-truth values of activity. This measure is an extension of previously introduced criterion calculated as the ratio of hit rate to false positive rate. Formally M1 can be written as

$M1=\sum_{i=1}^{n}|Jnor_{i}-\hat{J}nor_{i}|$ where normalized values of the ground-truth and estimated activations are calculated as $Jnor=J/\sum_{i=1}^{n}\left|J_{i}\right|$ and $\hat{J}nor=\hat{J}/\sum_{i=1}^{n}|{\hat{J}}_{i}|$.

Clearly, *M*1 is bounded and takes values in the 0–2 range with zero corresponding to the exact match and 2 to a complete absence of any overlap of the two solutions. *M*1 can be used without any threshold, however, its sensitivity gets then reduced due to taking into account low power but widely spreading tails. In this work we used a threshold to zero the activity of the vertices whose amplitude is below 10% of the maximum map value. The 10% threshold was considered as a reasonable number to use.

To asses the precision of activation timeseries recovery we used a very simple *M*2 correlation-based measure calculated as *M*2 = 1 − *r*, where *r* is the correlation coefficient of the recovered and the ground-truth timeseries. Note that M2 does not depend on the amplitude of this signal and reflects only shape similarity, as amplitude is essentially power characteristics taken care by *M*1. *M*2 is also bounded within 0–2 range with 0 corresponding to the exact shape coincidence and 2 to complete anticorrelation.

During systematic studies of GALA's behavior we varied neuronal activation parameters as described in the corresponding sections and generated several different random datasets for the same values of activity parameters. The number of such datasets for each parameter set varied in the 5–9 range depending on the complexity of computations. Values of *M*1 and *M*2 obtained for each of the cases were averaged and standard deviation was calculated. We have also averaged *M*1 and *M*2 values among subjects to make our presentation concise.

#### 2.2.2. Real data

In this work we used MEG data recorded during an experimental study in which the subjects within two 17-min sections were presented with 16 different visual stimuli. For testing our method on the real data we selected only two types of stimuli “face” and “scrambled face.” These stimuli were presented with all others in a randomized order for duration of 800 ms with an iter-stimulus interval that randomly varied in 1000–1500 ms range. Stimuli size measured 8° and all the stimuli were normalized to have the same brightness and RMS contrast. Stimulus “face” was a gray scaled image of a man's face processed with spatial bandpass filter with 0.025–0.1 band in the normalized spatial frequency units. The “scrambled face” stimulus was phase-shuffled, Fourier-transformed version of the “face.” Each stimulus was presented 50 times. During the experiment the subjects had to press a button in response to the target stimulus. Neither “face” nor “scrambled face” were the target.

MEG data was recorded with sampling rate 1000 Hz for eight middle-aged adult participants in a neuromagnetically-shielded room using a 306-channel MEG (Vectorview, Elekta-Neuromag) comprising 204 planar gradiometers and 102 magnetometers in 102 locations above the participant's head.

To avoid the concerns related to the different scales in the gradiometer and magnetometer data and to speed up the calculations in this methodological work we used only the data from 204 planar gradiometers for inverse solution testing. GALA could equally well be applied to the complete set of data combining gradiometer and magnetometer data.

The temporal signal space separation (tSSS) as implemented in MaxFilter (Elekta-Neuromag) was used to suppress interference signals generated outside the brain. The artifact corrected data were filtered with a 40 Hz low-pass filter. For each stimulus type the epochs comprising −100 to 600 ms relative to stimulus onset were extracted. This data was recorded in Moscow Center for Neurocognitive research (MEG Center) of Moscow State University of Psychology and Education. The study was approved by the ethics committee of Moscow State University of Psychology and Education.

### 2.3. Software notes

The main body of the software used in this study for simulations and for solving the inverse problem is custom designed Matlab software. To compute forward models we used Fieldtrip (Oostenveld et al., 2011) utilities. All operations related to canonical mesh creation and manipulation to match individual MRIs were performed using the functionality of SPM8 (Litvak et al., 2011) software package. For hyperparameter vector estimation we used SPM8 function implementing restricted maximum likelihood optimization.

## 3. Results

### 3.1. Simulation studies

The main idea behind GALA is the use of individual variability in position of anatomically and functionally identical cortical regions with respect to MEG sensors. Such variability may stem as from the individual anatomy variations as well as from varying head position against the sensors. We do not study these two sources of variation separately but rather start from individually computed lead field matrices (forward model matrices) for dipoles in the nodes of the canonical mesh. We then stack these individual matrices into the group forward field matrix as in Equation (2) and do so for 1,2,…, 9 subjects. For each such stacking we measure the effective rank of the group lead field matrix as the dimension of the principal subspace with normalized eigenvalues greater than *e*^−8^. As illustrated in Figure 1A there is a steady growth of the effective rank. Note, that for a single subject using 204 gradiometer sensors we observe the average rank to be 60 for *e*^−8^ condition number threshold and it demonstrates the 3-fold increase for 9 subjects.

We then simulated a singe time slice of activity in a single patch as shown in Figure 1C and projected on the sensors as described in Section 2.2.1. One and the same mesh was used to both generate the MEG data and to solve the inverse problem. However, the extent and shape of the Gaussian kernels was different for simulations and inverse problem solving. Since we aimed at comparing several methods (including non-iterative) we showed GALA results corresponding to the first iteration. The compared methods varied in using (or not) the similarity across subjects constraints and imposing (or not) the spatial smoothness constraint. See the Table 1 in for the complete list of methods used in this study.

**Table 1:** **Summary of the methods used in this study**.

**Inverse model type**	**Without smoothness constraint**	**With smoothness constraint**	
	**Non-interative**		**Interative**
No similarity across subjects	MNE	sMNE	sMNEi
	MNEave (MNE+post inverse averaging across subjects)	sMNEave (sMNE+post inverse averaging across subjects)	
Rigid similarity across subjects	Concat.	sConcat. equivalent model	Not used
Relaxed similarity across subjects	nsGALA	GALA(1)	redGALA
			(Model w/o source noise comp.)
			GALA

Figure 1B shows the dependence of *M*1 localization accuracy measure on the number of subjects in the group inverse. When lead field matrices are not concatenated the group analysis boils down to a simple across-subject averaging of the individually computed source maps. Although post-inverse averaging improves accuracy of localization of the local maxima (Larson et al., 2014), our results showed that averaging did not significantly improve M1 measure for both classical group-averaged MNE (MNEave) and MNE with additional spatial-smoothness constraint (sMNEave) (top two curves on the Figure 1B). Given that M1 is a combined measure reflecting both proximity of centroids for estimated and true activations as well as correct assessment of the extent of activated regions, we believe that the reason for this is that post-inverse averaging does not lead to more accurate estimation of activated area size.

The second from the top pair of nearly coinciding curves corresponds to a model similar to GALA but without smoothness constraint (nsGALA) and concatenated lead field based solution Equation (2) (Concat.). The two bottom curves correspond to full scale GALA after the first iteration (GALA(1)) and to the model equivalent to concatenated lead field based solution but with smoothness constraint (sConcat.). Actually, the results for the last model were calculated using the model similar to GALA but whose source space covariance does not have the individual variability term **D**^Θ^.

The observed coincidence of the curves in each of the pairs is expected since we have the exact patch coincidence across-subjects and therefore the contribution of the individual variability covariance component should reduce to zero. On the other hand, this observed behavior is not a trivial fact as this confirms the proper operation of the optimization step aiming to estimate from the data the hyperparameters **h** scaling the contribution of the two covariance components.

We also want to note that using the smoothness constraint in full scale GALA (GALA(1)) and model equivalent to concatenated lead field based solution with smoothness constraint (sConcat.) significantly increases localization accuracy (middle vs. bottom pair of curves). This was not the case for the standard approach when the smoothness constraint was applied individually to each subject (top two curves).

In order to appreciate the relation between the numerical values of *M*1 and the actual visually perceived similarity we present a set of maps with varying value of *M*1 produced by different inverse solvers (Figure 1C).

#### 3.1.1. The effect of non-exact spatial coincidence of functionally similar cortical structures

**Figure 4:**
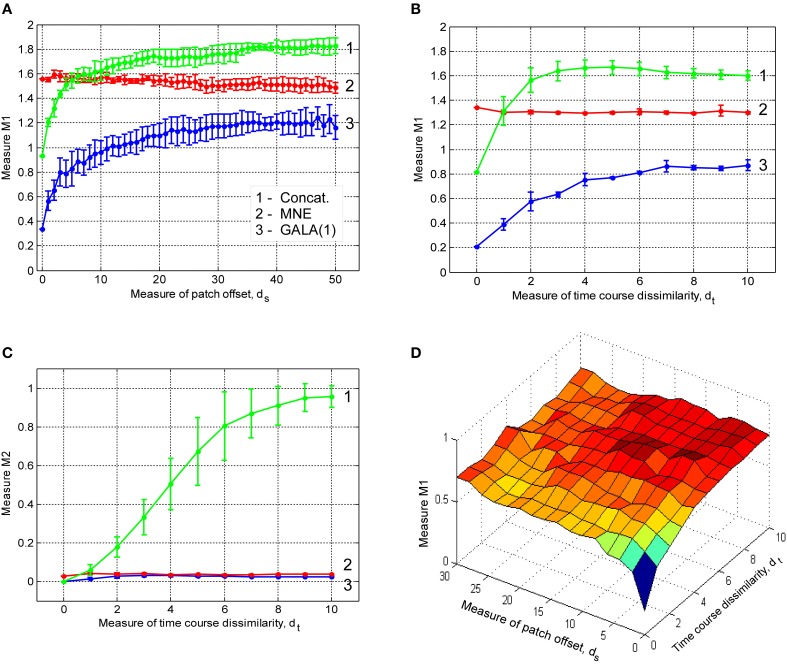
**Dependence of accuracy of ground truth activity recovery on the deviations from the exact across-subject similarity of activation vectors**. Dependence of *M*1 accuracy of ground truth activity recovery measure **(A)** on the spatial jitter of patch locations across subject (lower value of *M*1 corresponds to higher accuracy) for three patches simulated. When accuracy is assessed both in terms of spatial proximity of the estimated and true activations and correct specification of the extent of activated regions GALA(1) provides better accuracy than MNE for all simulated patch offsets. The model with lead-field concatenation (Concat.) performs worse than MNE even with a small displacement of patch centers. Dependence of *M*1 accuracy of ground truth activity recovery measure **(B)** and *M*2 **(C)** time course recovery accuracy measure on the dissimilarity of time courses across subjects for three different methods (lower values of *M*1 and *M*2 correspond to higher accuracy). Use numbers against each curve to establish the correspondence with the legend in (**A**). **(D)** Dependence of *M*1 accuracy of ground truth activity recovery measure on simultaneous patch centers displacement and activation timeseries dissimilarity. Localization accuracy provided by GALA(1) remains well bounded as revealed by almost horizontal dependence of *M*1 on simultaneous spatial and time jitter.

The results from the previous section illustrate that group inverse accuracy in the conditions of our study does not significantly increase for groups of more than 6 subjects. Therefore, to save on computer time we conducted the following studies using only the first 6 out of 9 alphabetically sorted subjects (which we believe is equivalent to a single random choice). We studied the effect of non-exact geometric coincidence of the central nodes of active cortical regions on the group inverse accuracy. We simulated three active patches per subject. We present our results as plots in Figure 4A. In these plots x-axis value *d_s_* is proportional to the average across-subject distance between patch centers. Detailed procedure used to determine patch locations for each of the subjects described in Appendix A.

The first thing that needs to be appreciated from the plots of *M*1 accuracy score in Figure 4A is that even with a small displacement of patch centers the lead field concatenated group inverse Equation (2) (Concat.) fails to deliver reasonable accuracy and performs worse than even the standard MNE solution. In contrast, for the entire range of simulated patch displacement values GALA(1) yields better M1 measure than that delivered by the MNE.

Based on the above we conclude that unlike the stacked lead-field inverse Equation (2) GALA permits a relaxation of the requirement of exact spatial coincidence of functionally similar cortical regions. GALA's accuracy of ground truth activity recovery exceeds that of the standard minimum norm approach even for very significant values of spatial jitter values. Primarily, this improvement is due to a more accurate GALA's estimation of region extents that is useful feature for disentangling different sources of activity in the three patch case.

#### 3.1.2. The effect of patch activation timeseries dissimilarity

In this part of our study we simulated three active patches with exact (node-by-node) spatial coincidence between subjects. We used different basic activation curves for each patch. We then used the procedure described in the Appendix to introduce across-subject variability of activation time-courses. Note, that the dissimilarity of activation time-course violates the assumptions implied by Equation (2). Interestingly, this leads not only to the errors in estimation of patch activation timecourses for the corresponding model (Concat.) (Figure 4C), which can be thought of as a trivial result, but also translates into significant deterioration of spatial accuracy as illustrated by *M*1 measure values in Figure 4B.

GALA's spatial accuracy deteriorates with increased dissimilarity of activation timeseries, however, it stays well below that of the standard minimum-norm and stacked lead field model based solutions. GALA(1) also provides nearly perfect accuracy of activation timeseries reconstruction for the entire range of dissimilarity values as opposed to the stacked lead field model.

The main conclusion here is that GALA as compared to the concatenated lead-field based inverse allows us to relax the requirement for exact similarity of patch activation timeseries across subjects. GALA also provides better reconstruction of the underlying source space activity mainly by means of more accurate estimation of activated area size than the standard minimum norm approach for all values of timeseries dissimilarity.

#### 3.1.3. The effect of simultaneous patch centers displacement and activation timeseries dissimilarity

In this section we describe our results of studying the effects of simultaneous spatial and temporal dissimilarity on GALA's accuracy. Figure 4D shows *M*1 measure accuracy surfaces corresponding to GALA(1) solution for three patch cases. We do not show the surface corresponding to the MNE solution as it appears to be a flat plane at, on average, *M*1 = 1.22 level for the three patch case studied here. We do not show *M*2 measure here as its values appear to be not significantly different from zero for both methods and cases studied in this section.

The surface in Figure 4D looks absolutely predictable based on the studies described in the previous sections. Under the exact similarity of temporal activations or zero patch displacement we are seeing the dependencies similar to those obtained in the previous sections where we scrutinized each of the effects separately. Noteworthy is the fact that for maximal values of each of the factors the accuracy looses its dependence on the other factor.

We conclude that for simultaneous variation of spatial and temporal similarity indices of the underlying source space activity GALA remains stable and produces solutions with accuracy superior to that of the standard MNE.

#### 3.1.4. The effect of iterations on the accuracy of the solution

**Figure 5:**
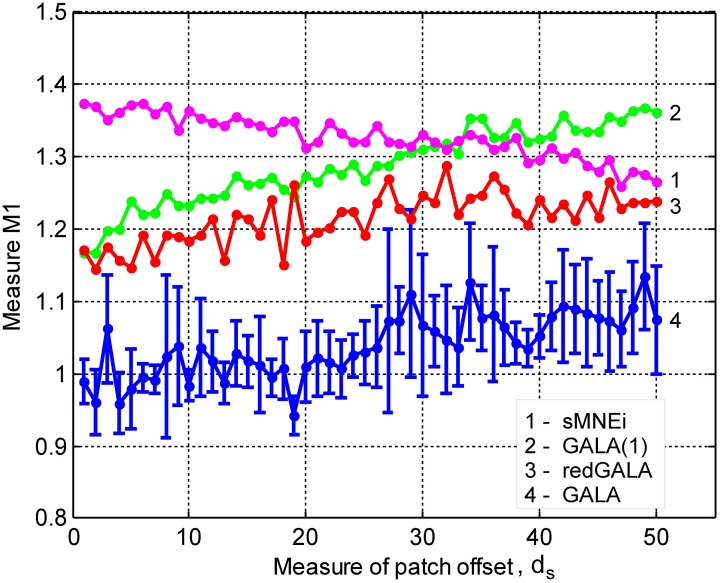
**Dependence of *M*1 accuracy of ground truth activity recovery measure on patch centers displacement for three different iterative algorithms (lower value of *M*1 corresponds to higher accuracy)**. Use numbers against each curve to establish the correspondence with the legend. The result provided by GALA after the first iteration (GALA(1)) are also shown. Errorbars are shown only for GALA after 6 iterations to avoid cluttering the figure. See the text for the description.

In this section we study the effect of iterations on the solution accuracy. We compare GALA with only one iteration (GALA(1)) to that of full blown iterative GALA for various degrees of spatial similarity of neuronal activity. We also study the effects of the use of source noise covariance component in GALA covariance Equation (11) on the accuracy and compare full GALA with its reduced version that does not use source noise covariance component (redGALA). We also compare the above to another iterative method based on the MNE with smoothness constraint (sMNEi) that loosely resembles FOCUSS-LORETA approach described in Liu et al. (2004). The smoothness constraint used by this method allows more accurate solutions to be obtained and represents a more challenging benchmark for GALA than the iterative method based on the standard MNE approach. Figure 5 shows *M*1 as a function of patch centers displacement for these four methods.

To simulate the evoked fields data we used three patches. We also added white noise to each vertex of the mesh providing *L*_∞_ amplitude SNR of 5 to model source noise in the evoked fields. As before, we then projected these noisy activations onto sensors using the forward field matrices.

First of all, as we can see from the relative displacement of blue (GALA) and red (redGALA) curves against the green curve corresponding to GALA(1), both GALA and reduced GALA increase performance with iterations. Interestingly, the effect of iterations on the reduced GALA approach is more pronounced for large spatial dissimilarity cases. Secondly, the use of source noise covariance components to model the non-common across-subjects activity significantly improves the localization accuracy. This can be seen by comparing the blue curve (GALA) and the red curve (redGALA).

Finally, we can see that GALA achieves significantly higher localization accuracy than the sMNEi algorithm. Note, however, that we do not use noise normalization for both algorithms and application of this technique could reduce the improvements furnished by GALA as compared to the sMNEi. Moreover the difference between these two methods tends to decrease with the increased spatial dissimilarity when GALA's assumptions of reasonable spatial similarity start being violated.

#### 3.1.5. Finding common across subjects cortical activity: realistic simulation of experimental conditions

**Figure 6:**
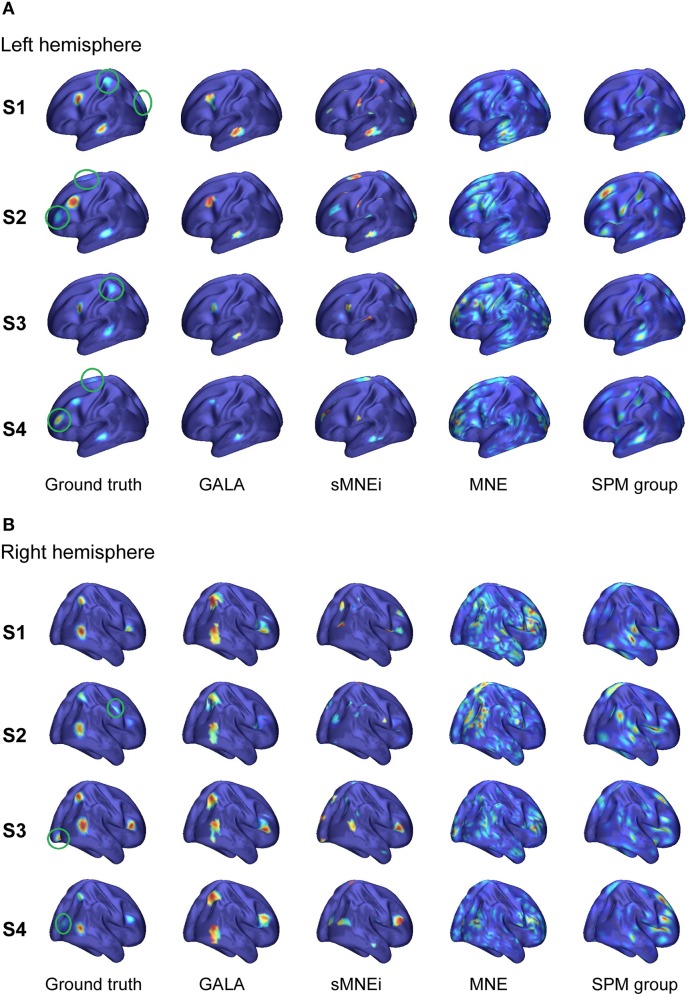
**Localization results for the realistic simulation of 5+3 active patches obtained with four different methods for 4 subjects S1–S4: (A) left hemisphere (B) right hemisphere**. Normalized activations for one time slice are shown. Green circles in the first column mark source noise patches. Note, GALA perfectly well finds all 5 patches with common for all subjects activity and discards source noise patches. sMNEi method converges to the local maxima of MNE, but these maxima do not match well with the simulated patches. SPM group inverse delivers a conditionally sparse solution represented by a set of common across subjects patches. However, the identified locations of these patches and distribution of their activity very significantly deviate from the ground truth.

This section describes our final simulations where we demonstrate the utility of GALA in recovery of specific and common across subjects activity under maximally close to realistic simulation conditions. We simulated data from 9 subjects with the common activity represented by 5 clusters as shown in Figure 6A. Patch centers for individual subjects were obtained by randomly shifting patch centers by 1–3 cortical mesh nodes with respect to the mean gravity node. We pseudo-randomly varied the shape of patch activation timeseries by means of temporal shift and spread to achieve the following average correlation coefficient values : 0.75 ± 0.08, 0.52 ± 0.13, 0.84 ± 0.05, 0.79 ± 0.07, 0.53 ± 0.1.

We used a cortical mesh with 8192 nodes to generate 5 active patches and projected their source space activity onto the sensors using the forward operator computed using single shell model. For inverse modeling we used a smaller mesh comprising 5124 vertices. We used smaller mesh for solving the inverse problem to mimic real-life situation when actual source positions may not coincide with either of the grid nodes.

We have also modeled non-specific to the task and individually variable activity using three additional patches of activity. To provide further physiological plausibility and reflect the fact that the primary source of this non-specific activity comes from the (unaveraged out) remains of ongoing activity whose spatial characteristics will have some similarity across subjects we have chosen those patches so that each patch is common to three out of nine subjects and that activity of these patches is significantly uncorrelated (*r* < 0.3). We would like to stress here that this situation with partly overlapping patches with non-specific (non-target) activity constitutes a significantly greater challenge to GALA than the case when spatial substrates of non-target activity do not coincide across subjects.

To model brain noise that remains in the averaged data we added 8196 noise sources (to each vertex of the canonical mesh). The activation time series of these sources were narrow-band signals obtained via zero-phase filtering of realizations of Gaussian (pseudo)random process by the fifth order band-pass IIR filters in the bands corresponding to theta (4–7 Hz), alpha (8–12 Hz), beta (15–30 Hz) and gamma (30–50 Hz, 50–70 Hz) activity. Their relative contributions were scaled in accordance with $\frac{1}{f}$ characteristic of the realistic EMEG spectrum. We added this noise to the clean source space data to obtain post-averaged source space *L*_∞_ SNR of 5. To model sensor noise we added to the sensor timeseries low-pass filtered (30 Hz cut-off) white noise and scaled it to obtain *L*_∞_ SNR of 3.3 in the sensor space.

Figure 6 shows comparative localization results for 4 randomly picked subjects (total 9 subjects were used in this simulation) for the following four methods (columns 2–5): GALA, iterative method based on MNE with smoothness constraint (sMNEi), the classical MNE and SPM group inverse. The SPM group inverse was chosen as the technique specifically designed to simultaneously solve the inverse problem for a group of subjects based on finding the common source space. This method may be used with various constrains imposed on the structure of the covariance matrix. We used the multiple sparse priors (greedy search) option to obtain a conditionally sparse solution comparable to that of GALA.

Table 2 shows goodness of fit, *M*1 and *M*2 performance measures for the four algorithms compared. Measure M1 was calculated for signal variance over the entire simulated time range. To compute this measure we downsampled the activity from the mesh with 8192 vertices to that with 5124 used for inverse modeling. For GALA we calculated M1 using only 5 common patches as by construction GALA infers this information from the data. For other methods the activity of all 8 patches was used to compute M1. This is due to the fact that these methods are simply not designed to find common across subjects activity and M1 calculated using only 5 patches would be necessarily worse. M2 for the MNE and SPM inverse solutions, due to their spatial continuity was computed using the vertices corresponding to the patches found by GALA. M2 for the iterative MNE model with smoothness constraint (sMNEi) can not be unambiguously calculated because it is impossible to establish the exact relationship between the original patches and those obtained by sMNEi solution.

**Table 2:** **shows goodness of fit (GOF, %), M1 and M2 performance measures for the four algorithms compared**.

**Method Measure**	**GALA**	**sMNEi**	**MNE**	**SPM group**
GOF (%)	56.92 (6.89)	86.36 (8.45)	90.78.11 (5.04)	58.22 (21.21)
M1	1.18 (0.08)	1.73 (0.14)	1.75 (0.0)	1.92 (0.09)
M2	0.09 (0.06)	–	0.09 (0.09)	0.63 (0.09)

*In brackets the standard deviations are shown. Measure M1 was calculated for signal variance over the entire simulated time range. To compute this measure we downsampled the activity from the mesh with 8192 vertices to that with 5124 used for inverse modeling. For GALA we calculated M1 using only 5 common patches as by construction GALA infers this information from the data. For the other methods the activity of all 8 patches was used to compute M1. This is due to the fact that these methods are simply not designed to find common across subjects activity and M1 calculated using only 5 patches would be necessarily worse. M2 for the MNE and SPM inverse solutions due to their spatial continuity was computed using the vertices corresponding to the patches found by GALA. M2 for the iterative MNE model with smoothness constraint (sMNEi) can not be unambiguously calculated as it is impossible to establish the exact relationship between the original patches and those obtained by sMNEi solution*.

SPM group inverse with multiple sparse priors delivers conditionally sparse solution (each patch's extent is formally infinite but has Gaussian shape and can be thresholded) with common across all subjects patches. Localization of these patches poorly matches the ground truth (Figure 6) as reflected in a very high value of *M*1 = 1.92 for this method. In addition, SPM group inverse with MSP option corrupts the temporal structure of response. Average across subjects *M*2 = 0.63, that is significantly higher than for GALA or MNE. This result can be explained by the fact that due to a poor match between the ground truth and SPM detected patches *M*2 was calculated, in fact, using the timeseries of vertices falling in between the centers of these patches, and therefore appears to be superposition of patch low-amplitude tails. Also, it appeared to be impossible to unambiguously match the SPM obtained and ground truth patches. Note that contrary to GALA, that operates on the joined source space and effectively increases forward matrix rank, SPM group inverse seeks the intersection of the source-space from different subjects and therefore reduces the effective rank in the data.

The standard MNE solution is distributed over the entire cortex and its interpretation is complex primarily due to dependence of the resultant ROIs and their activation timeseries on the thresholds used. Local maxima of the MNE solution do not correspond well with the simulated patches (and patches found by GALA). However, it is interesting to note that the MNE timeseries calculated for patches determined by GALA show nearly perfect reconstruction. Also, note that we show here the results provided by MNE without applying any form of noise normalization or statistical techniques. The use of these post-inverse steps could potentially improve the accuracy of the MNE estimated activity maps.

Thus, we have demonstrated that when modeling assumptions are fully satisfied GALA allows near perfect recovery of the spatial and temporal structure of the common to all subjects neuronal activity. GALA also takes care and excludes from the solution the activity that does not exhibit fuzzy similarity across subjects. For the other 5 subjects localization is qualitatively similar. Sparse localization provided by GALA allows for the exact coregistration of the discovered patches across subjects. Comparing GALA with the other iterative method used in this simulations (sMNEi) we can see how the sparseness (a desirable property on one hand) of the solution provided by this approach makes it difficult to establish the correspondence of functionally equivalent cortical regions across subjects. Patches found using sMNEi method do not overlap and the count of such patches varies from subject to subject.

### 3.2. Application to real data

**Figure 7:**
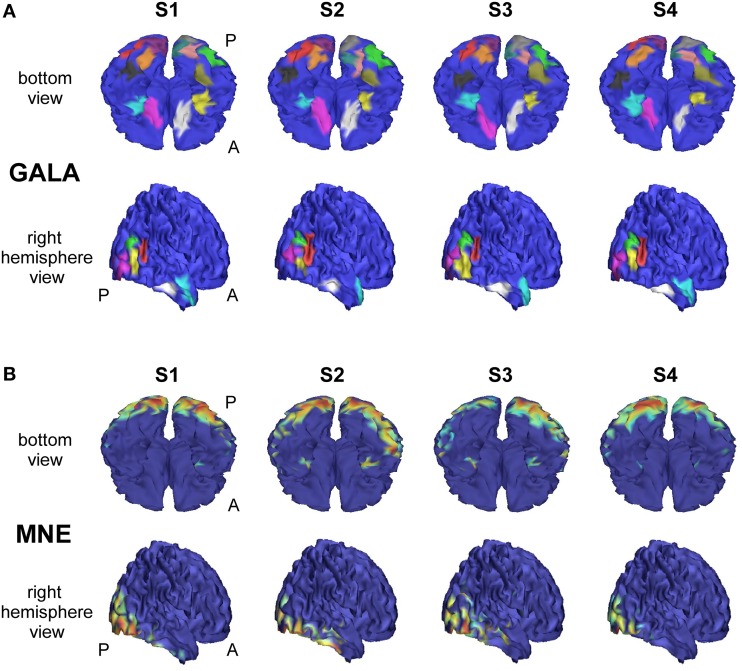
**Localization results for the real data recorded during presentation of “face” and ”scrambled face” visual stimuli obtained with two different methods for 4 subjects S1–S4**. **(A)** GALA results: color-coded patches of activity common to all 8 subjects—the same color codes corresponding pathes across subjects. These patches exhibit a great deal of overlap across subjects, but sometimes have considerable shifts and shape variations. **(B)** MNE results: variance of the solution over the post-stimulus 0–600 ms time range thresholded at 0.9 of the maximum value. To the first approximation, patches found by GALA coincide with activity peaks obtained by the MNE. However, the activity in the fusiform (FFA) area (GALA's black and khaki patches), that is especially important in our experimental paradigm, is not readily noticeable in the MNE solution for any of the subjects for the chosen threshold. *A* denotes anterior part of the brain; *P*—posterior part.

We analyzed an MEG dataset comprising data from 8 subjects as described in Section 2.2. We used GALA and MNE techniques and compared the results delivered by these two methods. Figure 7 shows localization results obtained using GALA for four randomly chosen subjects (Figure 7A) and the MNE (Figure 7B). For GALA we show color-coded patches of activity common to all 8 subjects. For the MNE we show variance of the solution over the post-stimulus 0–600 ms time interval thresholded at 0.9 of the maximum value. This threshold was chosen to ensure approximately equal number of mesh vertices highlighted by GALA and the MNE solutions. For GALA we performed 3 iterations. This number was chosen to match the goodness of fit (GOF) delivered by GALA to that of the MNE — 94.32 vs. 93.12 correspondingly. High value of GOF does not guarantee the actual goodness of the solution (see for instance, Table 2 in the previous section). However, in order to make the results of the two methods comparable we have chosen to match them based on the correspondence to the measured data using the GOF measure.

After 3 iterations GALA found 23 functionally equivalent ROIs common for all subjects. The correspondence between regions of different subjects was automatically established by clustering based on the metrics that combines geodesic distance on the mesh and across subject correlation of activation timeseries calculated in local maxima of 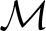
*_i_* for each *i*-th subject. GALA's property of keeping the low activation magnitude of vertices corresponding to the non-common part of the solution significantly facilitates the clustering process.

As we can see from Figure 7A these regions exhibit a great deal of overlap across subjects, but sometimes have considerable shifts and shape variations. Interestingly, the ROIs found on the basal surface show nearly perfectly symmetric structure although the algorithm did not have implicit constraints stipulating any kind of symmetry. On the lateral surface such symmetry is not present, and the right hemisphere is characterized by a more pronounced activity that finds its reflection in greater count of ROIs and higher amplitude of activations.

To the first approximation ROIs found by GALA coincide with activity peaks obtained by the MNE. However, this is only true for areas with the most pronounced activity. We have purposefully chosen an experimental paradigm where the most specific and thus the most interesting activity is not easily defined by high amplitude of activations. Considerable evidence from behavioral, neuropsychological and neurophysiological investigations support the hypothesis that humans have a specialized brain region—fusiform face area (FFA)—dedicated to the perception of faces (see for the review Kanwisher and Yovel, 2006). But the amplitude of FFA activation is significantly lower than the amplitudes of more primary visual responses arising in occipital pole (OP) and lateral occipital complex (LOC). And we can see that the activity in the fusiform (FFA) area is not readily noticeable in the MNE solution for any of the subjects for the objectively chosen threshold. The use of some form of noise normalization or within-subject statistics could potentially amend the situation. In contrast, GALA without applying any form of noise normalization found two symmetrical ROIs for each subject in the expected locations corresponding to the left and right FFA.

**Figure 8:**
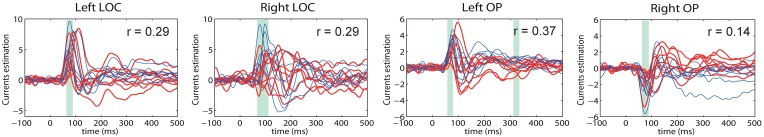
**MNE estimated timeseries for MNE defined ROI**. Red lines—timeseries for 8 subjects in “face” condition; blue lines— timeseries for 8 subjects in “scrambled” condition. The cyan shadow stripes mark time intervals with duration greater than 20 ms and with values of *t*-statistics corresponding to 0.05 significance level of uncorrected *t*-test. No time intervals with *p_corrected_*(*t* < 0.05) were found. We used *t*-statistics here to quantify the observed distance between conditions and by no means as a statistical test aimed at finding intervals of significant differences. Mean across subject correlation coefficients for each ROI are shown. Note, that ROIs selected based on the MNE solution give rise to quite different activations of the symmetric cortical regions. LOC, lateral occipital complex; OP, occipital pole.

The main advantage of our new method, however, can be appreciated via comparative analysis of activation timeseries delivered by the standard MNE and GALA approaches. Let's first consider ROI timeseries derived from the MNE solution. Following the standard practice we created four ROIs corresponding to the global maxima of the MNE detected activity averaged over all subjects. These ROIs appeared to be located in the left LOC, left OP, right LOC and right OP cortical structures. The actual vertices forming these ROIs were found in a standard way by thresholding the averaged across subjects MNE solution. ROI timeseries were taken to be the first right singular vector of the ROI vertices timeseries matrix. Figure 8 shows MNE ROI timeseries for the two different experimental conditions. As we can see these timeseries show quite variable activations that result into low correlation coefficients in the 0.14–0.37 range. This within condition variation obscures the difference between experimental conditions. We measured this difference using the *t*-statistics calculated for each time slice and used it purely to quantify the distance between the responses observed in the two conditions.

**Figure 9:**
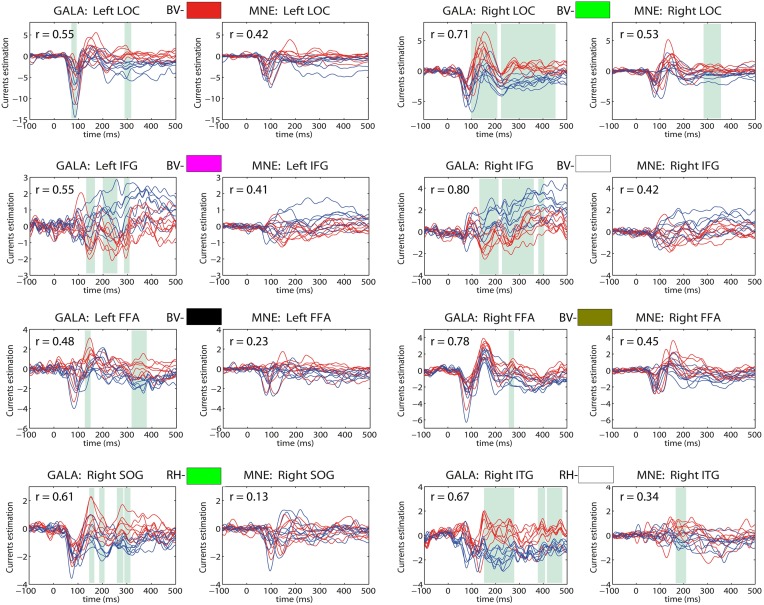
**Estimated timeseries for GALA defined ROIs: columns 1 and 3—for GALA estimated timeseries; columns 2 and 4—for MNE estimated timeseries**. Colored rectangles above each pair of each ROI timeseries establishes the correspondence with colored ROI patches in Figure 7. BV stands for bottom-view and refers to the ROI with the corresponding color in the bottom view, RH similarly corresponds to the right hemisphere view. Red lines—timeseries for 8 subjects in “face” condition; blue lines—timeseries for 8 subjects in “scrambled” condition. The cyan shadow stripes mark time intervals with duration greater than 20 ms and with values of t-statistics corresponding to 0.05 significance level of (Bonferroni) corrected *t*-test. We used *t*-statistics here to quantify the observed distance between conditions and by no means as a statistical test aimed at finding intervals of significant differences. Mean across subjects correlation coefficients for each ROI are shown in the top-left corner of each plot. For GALA estimated timeseries these coefficients are significantly higher than for the MNE estimated timeseries for each chosen ROI. Note also, that symmetric brain regions (first three rows) are now served by more similar than in the MNE case timeseries. LOC, lateral occipital complex; IFG, inferior frontal gyrus; FFA, fusiform face area; SOG, superior occipital gyrus; ITG, inferior temporal gyrus.

In contrast, Figure 9 shows timecourses for 8 most functionally and anatomically relevant ROIs in the context of the task performed whose exact location was found with GALA. Columns 1 and 3 show GALA derived timeseries based on GALA defined ROIs and columns 2 and 4 correspond to patch timeseries computed for the same GALA defined ROIs but using MNE calculated vertex timeseries.

We can see that more accurate ROI borders discovered by GALA lead to significantly increased across-subject similarity of the corresponding timecourses even for MNE calculated ones. We can now observe significantly higher average correlation coefficient values, for example, we see 0.42 and 0.53 for the left and right LOC correspondingly. Also, symmetric brain regions are now served by more similar timeseries than in the case of MNE discovered ROIs with matching peak latencies and polarities of the half-waves. While the similarity of the timeseries in the corresponding ROIs is enforced by our algorithm according to its very design no symmetry related constraints are imposed. Therefore, we believe that this observed symmetry of activations in homologous symmetric structures is not a trivial result and contributes to the physiological plausibility of the solutions obtained with GALA.

The full blown GALA solution gives even higher similarity as can be appreciated by comparing the correlation coefficients for each pair of plots corresponding to the same ROI. Interestingly enough the *t*-statistics based distance between activations observed in the two different conditions is noticeably greater in GALA derived solutions. Noteworthy, is also the fact that the increase of such a distance is observed not in all but only in physiologically relevant regions as discussed above. Therefore, we believe that this increased distance comes from both the reduced within condition variance entrained by GALA and the genuine difference between the two conditions. To render statistical significance estimates to such difference a proper statistical test is needed that would take into account the mutual dependence of individual solutions derived by GALA. We discuss this issue further in the next section.

The main conclusion here is that even this partly sparse GALA's solution can provide an easy way to detect common across subject ROIs characterized by similarity of both spatial location and temporal behavior. This data-driven way for detection of ROI borders is free from arbitrariness encountered with *apriori* ROI definition. Using similarity across subjects constraint during inversion and the extended criterion for best vertices (using not only variance) selection can find ROIs that are not readily noticeable in traditional approaches based on amplitude thresholding.

## 4. Discussion

The main motivation for development of the GALA approach was not so much the desire to create a new technique for solving the MEG inverse problem, but rather the need to address a broader question and develop a framework for exploratory time-resolved analysis of group MEG data. Usually, cross-subject differences in cortical surface geometry and head position with respect to the helmet make it difficult to recover task specific activity. This happens primarily due to the difficulty in locating and co-registering functionally identical activity across-subjects (Henson et al., 2011; Bigdely-Shamlo et al., 2013; Jafarpour et al., 2013). In this work we extended the observation originally reported in Larson et al. (2014) and used these cross-subject differences to our advantage. We treated these variation as additional information for the group inverse solution instead of at the post individual inverse stage and demonstrated the possibility to obtain significantly more accurate and more interpretable solution of the MEG inverse problem.

The solutions obtained with GALA appear to be situated midway between those derived by fully distributed (imaging) and focal (dipolar) source models. By first assuming the continuity of cortical activity distribution, GALA results in a sparse set of patches with clearly specified boundaries and temporal activations. In addition, GALA by design facilitates the cross-subject correspondence between found ROIs. This way GALA combines the pros of these two principally different approaches and avoids their cons.

We would also like to stress that by relaxing the requirement of complete overlap of the corresponding patches GALA allows for a significant statistical advantage over more standard SPM-based methods in determining the cortical regions differentially activated under experimental task manipulation. Indeed, the tests employed by the SPM approach appear to be of massively univariate nature and accomplished on a vertex-by-vertex basis comparing the corresponding vertices across individuals (Friston et al., 1991). This approach implicitly assumes the exact functional coincidence of anatomically matched ROIs and looses its statistical power when this assumption is only partially correct. Partly, this can be illustrated by comparing the timeseries, the result of the MNE procedure, presented in Figure 8. We can see that this assumption results into the inability to discern the two conditions and see reliable difference in activations. In contrast, using the ROIs discovered by GALA similar analysis allows to detect expected under this experimental paradigm differential activation in the right LOC area, see Figure 9. The only difference between the two approaches is the use of more accurately detected ROI boundaries performed by GALA and based not only on anatomy but also using functional information present in the data. GALA was applied to the two conditions simultaneously and aimed to match the activation timeseries of subjects in each of the two conditions without forcing the difference between the conditions. Therefore, the non-rigid alignment of ROIs performed by GALA may be considered as a removal of systematic error in the data that consequently should lead to the improved power of the statistical test.

However, the standard statistical procedures can not be applied to the individual source maps derived by GALA due to their statistical dependence. Consider the following mental experiment. Assume that we have a set of vector observations obtained under two different conditions. The vectors thus have two different sources of variation—between observations (between subjects in our case) and between conditions. Now, imagine that the subspace in which these vectors vary between observations is orthogonal to the subspace in which between condition variations take place. In this case, if we somehow accounted for this within condition (between observations) variation we will still have the same amount of between condition variation left. Additionally, the reduction of the systematic (between observations within condition) variations will actually increase the power of the subsequent between conditions test. In real life we can not guarantee the orthogonality of these two subspaces and therefore accounting for the systematic variations across subjects leads to the reduced independence of observations and needs to be treated with a special significance correction coefficient whose value depends on the overlap between the two mentioned subspaces. This correction coefficient will be a function of the residual (unexplained) variance and the degree of similarity of individual solutions obtained by GALA. Derivation of the exact expression for this correction coefficient is the subject of our future efforts. Another possibility is to treat the multi-subject dataset as a single subject but with multiple heads and follow randomization test strategy randomizing condition labels on the individual trials level.

The average running time (204 Gradiometers, 2 conditions, 9 subjects, 700 timeslices, 5124 vertices, 5 iterations) of GALA is approximately 12 min. We believe that this machine time favorably compares to the solution quality obtained and to the amount of time spent by the researcher identifying the across-subject correspondence between the active patches detected by a conventional algorithm.

This paper is a first introduction to this new method, and thus aims primarily to emphasize the main ideas behind GALA. GALA, to a simple approximation, is a variant of classical MNE that explicitly takes advantage of the increased rank of lead field matrix. Therefore, here GALA is compared only with standard MNE, without noise normalization or other statistical methods employed, despite the fact that these methods may improve MNE localization accuracy. To fully understand GALA and compare its performance to MNE, future work is necessary to investigate GALA and MNE in various noise conditions, using different forms of noise normalization and statistical techniques that could potentially improve both GALA and MNE.

Taken all together the characteristics of solutions obtained by GALA make them ideal candidates for subsequent connectivity analysis (Lachaux et al., 1999; David et al., 2006; Schogl and Supp, 2006; Wibral et al., 2011; Greenblatt et al., 2012), providing the essential prerequisites, namely, accurately derived timeseries and exact ROIs matching. Importantly, ROIs found by GALA do not necessarily have activity whose power characteristics vary across experimental conditions. The ROIs found by GALA (and their activity) aim to explain the experimental data in both conditions and thus do not necessarily correspond to differentially activated cortical regions. The absence of such differential activation does not exclude the possibility for such ROIs to be a part of a functional network with connectivity properties modulated by the experimental task manipulation. Therefore, in contrast to more standard approaches for selection of ROIs to be used in connectivity analysis, ROIs discovered by GALA allow for a more comprehensive connectivity analysis based on the functionally significant (but not necessarily differentially activated) ROIs. To be able to perform connectivity analysis GALA in the future will be extended to work with single trial data. One way to do this is to use activation similarity metrics based on the power spectral density of ROI activations. Another way is to use the inverse operator along with ROI masks derived by GALA iterations and apply it to single trial data to compute connectivity measures.

Another direction for our future efforts is to develop an objective criterion for dealing with reparametrization of the basis set of covariance components and termination of iterations. As we showed, each iteration reparametrizes the basis set of covariance matrices. This reparametrization is done based on the metrics calculated using non-diagonal elements of the covariance matrix. To replace this heuristics we are going to exploit the results of Friston et al. (2008a,b) showing that for a simple form of reparametrization like ARD or Greedy search the best solution can be chosen maximizing the same objective function as the one used for hyperparameters estimation. We are now in the process of testing whether this approach is capable of operating in more general situations and in particular for significantly non-diagonal covariance components imposing similarity across subjects constraints. In this work we stopped the iterations either based on the knowledge of the ground truth for simulated data or, when working with real data, based on the GOF values similar to those delivered by the standard MNE solution. Alternatively, prespecified number of active cortical regions uniquely matched between subjects could be used as a stopping criterion.

Clearly, the extent of improvement in solving the inverse problem delivered by GALA is conditioned on the accuracy of the individual forward models with the latter dependent on the fidelity of individual cortices representation. This requirement is especially crucial for forward models using cortically constrained orientations of vertex dipoles as inaccuracies in dipole orientations may cause greater errors in forward models than errors in dipole location parameters (Salayev et al., 2006). The canonical mesh technique used in this work to guarantee one-to-one correspondence between source spaces of individual subjects only allows to establish a coarse correspondence between the geometric parameters of different brains and matches location of main sulci and gyri only, leaving the subtle structure and individual cortical folding patterns unaddressed. Therefore, another potential direction to improve GALA's accuracy is to use more realistic individual meshes obtained using more sophisticated techniques than those exploited in the present study.

### Conflict of interest statement

The authors declare that the research was conducted in the absence of any commercial or financial relationships that could be construed as a potential conflict of interest.

## Appendix

### Simulating anatomical variation of patches

To simulate subject-to-subject variation of anatomical structure we varied the positions of ROIs. More precisely, the value patch offset marked on x-axis of the corresponding plots in Figures 4, 5 is calculated as follows. Null corresponds to the exact coincidence of patch centers across subjects, i.e., unshifted patch centers. To understand the procedure when applied to three patches, first, consider a single patch. In this case, *d_s_* = 1 corresponds to a shift of the first subject's patch center to 1 unit over mesh adjacency metrics with respect to the common for all subjects and patches center of mass. Increment of *d_s_* by 1 leads to the shift of the second subject's patch center and so on until *d_s_* becomes equal to the number of subjects (*d_s_* = 6 in our case) and all subject's patches are shifted by one unit. Then, further increase (*d_s_* = 7) leads to shifting first subjects patch center by one more unit, resulting in total shift of 2 units along the mesh adjacency metrics with all the other subject patches remaining shifted by 1 unit. Pseudo-random distribution of the vertices chosen results into uniform distribution of the shifted patches around the center of mass providing for (on average) a linear growth of the distance between them.

In the demonstrated three patch case we follow the same ordered shifting scheme with addition that all the patches of the first subject have to be shifted (by one unit) before the first patch of the second subject can be moved by one unit. In this case, maximum value of *d_s_* = 50 means that in four subjects each patch is shifted by 3 units, one subject has 2 patches shifted by 3 and one patch shifted by 2 units and all patches of the last subject are shifted by 2 units. Average distance between the centers of the corresponding patches is equal to 2.85 units.

The average geodesic distance between the adjacent vertices in the cortical meshes used for this simulation was 6.9(±3) mm. The values reported in mesh units can easily be recalculated. For instance the average distance between the centers of the corresponding patches is asproximately 20 (±9) mm.

### Simulation of activation timeseries variation

To gauge and simulate the across-subject dissimilarity of activation timeseries we used measure *d_t_* that is used along x-axis in Figure 4. When *d_t_* = 0 we have exact similarity of individual activation timeseries. From here patch timecourses of all but one randomly chosen subject are shifted forward in time and additionally spread out by the amount proportional to the ordinal number of the subject multiplied by *d_t_*. This procedure is applied to each patch separately. Thus, *d_t_* is not a linear function of the average across-subject correlation coefficient of timeseries. Nevertheless, *d_t_* is a monotonically decreasing function without deflection points. In our case *d_t_* = 10 corresponds to average correlation coefficient of 0.18 which corresponds to a very low degree of timeseries similarity and thus, the corresponding simulated data allow us to test the robustness of the algorithms to violation of the assumption about similarity of temporal activations for homologous brain regions.
